# Adding a parent-focused child sexual abuse prevention module to home visiting: a provider cohort study protocol

**DOI:** 10.3389/fpubh.2026.1819565

**Published:** 2026-06-03

**Authors:** Ella Abourjaily, Vanessa Abuchaibe, Alex Dahlen, Shannon Self-Brown, Roddey Jones, Allison Kemner, Kate Guastaferro

**Affiliations:** 1 Department of Social and Behavioral Sciences, New York University School of Global Public Health, New York University, New York, NY, United States; 2 Department of Biostatistics, New York University School of Global Public Health, New York University, New York, NY, United States; 3 National SafeCare Training and Research Center, Georgia State University School of Public Health, Georgia State University, Atlanta, GA, United States; 4 Parents as Teachers National Center, St. Louis, MO, United States

**Keywords:** child sexual abuse, home visiting, implementation fidelity, parent education, primary prevention, providers

## Abstract

**Background:**

Home visiting programs are an effective resource for parents to learn how to best support and protect their children as they grow and develop; however, there is a gap in home visiting content for equipping parents with the knowledge and skills needed to protect their children from child sexual abuse (CSA). Delivering CSA prevention content may be unfamiliar or uncomfortable, even for seasoned home visiting providers (i.e., parent educators). As provider acceptability and self-efficacy are critical predictors of implementation fidelity and the effectiveness of any added module, it is essential to evaluate these factors accordingly. This study is nested within an ongoing national clinical trial (NCT05976867) assessing the effectiveness of adding a CSA prevention module to Parents as Teachers, an evidence-based home visiting model. Leadership at participating sites (*N* = 25) will complete a baseline survey to aid in understanding providers’ environmental contexts.

**Methods:**

Providers who agree to participate in research (target *N* = 100) will complete longitudinal assessments evaluating changes in attitudes and self-efficacy from baseline (pre-training) to immediately following training in the CSA module, and annually thereafter (up to 3 years). The primary modeling approach will use linear mixed-effects regression to fit the outcomes attitudes and self-efficacy. Annual semi-structured interviews will be analyzed qualitatively both deductively and inductively with a codebook centered on study aims and the REAIM framework.

**Discussion:**

This work will contribute to understanding implementation practicalities, provider acceptability, and key areas in which to strengthen provider training to bolster implementation fidelity and ultimately promote the effectiveness of the CSA prevention module.

**Ethics:**

The procedures described herein were approved by the New York University Institutional Review Board (FWA00006386). Informed consent will be collected from all participants who wish to participate in the study.

**Dissemination:**

Upon study completion, the research team anticipates that findings will be disseminated to academic peers through publication and shared with the Home Visiting Applied Research Collaborative (HARC).

## Highlights

- Asking providers to deliver content related to sensitive content, like child sexual abuse (CSA), may impact implementation fidelity
- Longitudinal assessment of provider attitudes and self-efficacy allows us to understand implementation practicalities and ways to strengthen provider training
- Studying implementation fidelity will help to examine the effectiveness of the CSA prevention module
- Lessons from this study may be applied to other efforts in the home visiting space exploring enhancement to curricula

## Introduction

### Background and rationale

Child sexual abuse (CSA) is a preventable public health problem, yet recent estimates demonstrate a prevalence rate of 21.7% in the US (1, 2). All children are at risk for CSA and costly biopsychosocial consequences are often lifelong (3–5). When equipped with protective behavioral skills, parents are uniquely positioned to protect their children from victimization (6, 7). Home visiting programs designed to support parents of children from birth to five-years old have made strides toward preventing subtypes of child maltreatment including physical abuse and neglect (8–11) and are supported by federal funds [e.g., Family First Prevention Services Act (FFPSA) and the Maternal Infant Early Childhood Home Visiting (MIECHV) Program]. However, CSA has remained largely unaddressed in the content of home visiting. This is primarily attributable to home visiting programs’ foci on (a) promoting the parent–child relationship and (b) modifying parent behaviors through education on child development (12, 13). Because the etiology of CSA is complex and differs from that of other maltreatment subtypes, CSA prevention requires unique intervention that rather centers on the child’s social and physical environment (6, 14, 15).

There is a dearth of parent-focused CSA prevention interventions – specifically those that take the burden of prevention away from the child. However, results from those that exist have shown promising gains in parents’ CSA-related knowledge, behavioral intentions, and self-efficacy (16). Provided the positive impact that evidence-based home visiting programs have demonstrated in the prevention of physical abuse and neglect, several calls have been made for the inclusion of CSA prevention content to be incorporated into home visiting models (6, 17). Home visiting models are well positioned to deliver CSA prevention to parents given that foundational parenting skills are taught concurrently, increasing efficiency and cost-effectiveness (12, 18). In addition, incorporation into home visiting encourages expansive reach of selective parent-focused CSA prevention. Not only do home visiting programs serve upwards of 280,000 families in the US annually with weekly, biweekly, or monthly visits (19), but they also serve families that are at elevated risk given engagement with and referral by child welfare services (20, 21). To the best of our knowledge, only one intervention has been developed addressing the call to include CSA prevention content in home visiting models: Smart Parents – Safe and Healthy Kids (SPSHK) (16, 22, 23).

#### Smart Parents – Safe and Healthy Kids

SPSHK is a selective, evidence-informed CSA prevention intervention tailored to parents enrolled in home visiting (22, 23). The program is designed to be delivered in one typical home visit (60–90 min) by a trained and certified provider. Rooted in social learning theory, the SPSHK curriculum builds off of the content of home visiting programs to include CSA-specific content related to: (a) healthy child sexual development; (b) parent–child communication about sex and sexual development; and (c) child safety in the home, outside the home, and online. In the SPSHK session, providers (i.e., home visitors, parent educators) lead parents through a series of role-play scenarios used to explore potential situations, model preventive behaviors, and practice formulating age-appropriate responses, before providing positive constructive feedback. The goal of SPSHK is to increase parents’ knowledge and awareness about CSA as well as their use of preventive behaviors to protect their child(ren) from victimization. Preliminary evidence for SPSHK has demonstrated significantly greater CSA-related awareness (*p* < 0.001) and intentions to use preventive behaviors (*p* < 0.001) among parents who received SPSHK as an added module in their home visiting curricula compared to those who did not, while maintaining the original efficacy of the home visiting program (22).

#### Delivery of SPSHK by home visiting providers

There is an opportunity to deliver effective CSA prevention content as a component of evidence-based home visiting programs that currently focus on child physical abuse and neglect prevention. However, it is important to consider that the delivery of content pertaining to CSA prevention may be perceived as novel or discomfort-inducing for home visiting providers (e.g., parent educators), due to the sensitive and complex nature of the topic. As provider acceptability and self-efficacy are critical predictors of implementation fidelity (24–26), it is essential to the effectiveness of the intervention that they are evaluated accordingly. A pilot study designed to understand feasibility and acceptability of SPSHK as an added module to home visiting models demonstrated positive findings (23). Providers (*N* = 9) initially expressed concerns about adding another module to their already full curriculum, specifically considering the topic of CSA, sharing skepticism about whether it would be a good fit. After viewing delivery of the curriculum, however, perceptions of feasibility and acceptability among providers appeared to shift positively wherein providers felt the session length was long, but agreed that SPSHK would be highly beneficial for parents and aligned well with their existing home visiting curriculum.

A preliminary study designed to further explore provider attitudes and beliefs after being trained to deliver SPSHK aligned with the pilot findings (27). Prior to training, a naïve sample of providers (*N* = 33) shared initial concerns about discussing sex and sexual abuse with parents on their caseloads, given the sensitivity and perceived taboo of the topic. Others communicated additional hesitancies citing time management, the potential for parent resistance, and a lack of confidence in their ability to disprove myths. Post-training, significant increases were found in provider acceptability and self-efficacy both immediately (Mean *Δ* = 0.6) and after 6-months of implementation (Mean Δ = 0.5) (27). These findings hold promise for future dissemination of parent-focused CSA prevention, however, evaluation of provider attitudes and self-efficacy at a larger scale is needed to bolster evidence and to further inform training and implementation practices. Expanding this evaluation to explore implementation outcomes guided by the RE-AIM framework (i.e., reach, effectiveness, adoption, implementation, maintenance) (34, 35) with particular attention to adoption, implementation, and maintenance, would contribute to a more comprehensive understanding of provider and site readiness and inform future dissemination, sustainability, and scalability practices.

### Current study

The current study is nested within the Smart Parents Study (R01 HD108209), a national hybrid implementation-effectiveness trial designed to evaluate the addition of SPSHK to Parents as Teachers (PAT), an evidence-based home visiting program. PAT is delivered by paraprofessionals with a preferred minimum level of education attainment at the associate’s degree level (28). All parent educators (i.e., providers) complete a 40-h training before they begin to work with families and engage in 20 h of continued education annually (28). In our prospective, longitudinal, cohort study, parent educators at the *N* = 25 PAT Affiliate Sites engaged in the Smart Parents Study (target *N* = 100) will be followed over 4 years. The aims are to evaluate the evolution of provider attitudes and self-efficacy throughout the study period (Aim 1) and to explore how contextual differences (i.e., site and provider characteristics) moderate provider attitudes and self-efficacy (Aim 2). Results will further the understanding of SPSHK implementation practicalities and acceptability, as well as knowledge of how to best support providers in training them to deliver parent focused CSA-prevention. Ultimately, this will inform future steps to ensure implementation fidelity and promote the effectiveness of the intervention, equipping parents with accurate knowledge and skills to protect their children from victimization.

## Methods and analysis

### Study design and participants

**Figure 1:**
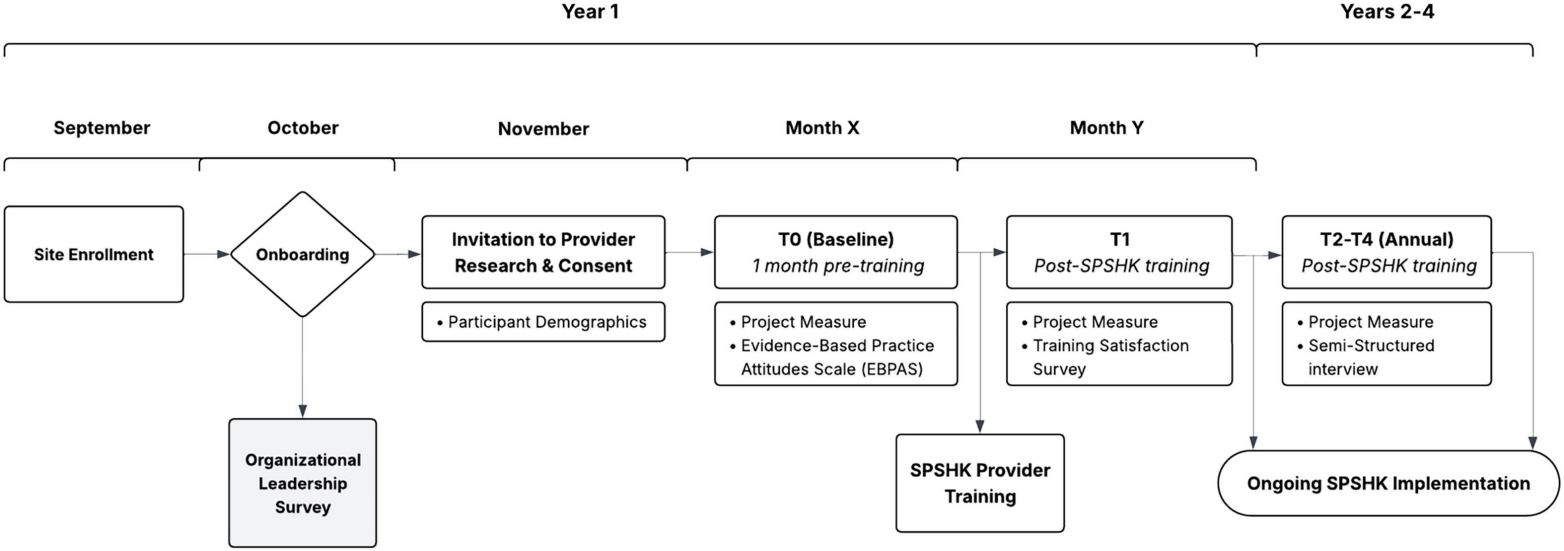
Study timeline. Provider training will be staggered to follow the stepped-wedge cluster design of the smart parents study. See Table 1 for Month X and Month Y dates by Step.

The overall Smart Parents Study follows a stepped-wedge cluster randomized control design, a pragmatic experimental design wherein randomized clusters (i.e., sites) sequentially transition from control to intervention until all clusters are exposed to the intervention (i.e., SPSHK). Sites (*N* = 25) were randomized into 1 of 5 steps which dictated the order in which they would be trained to trained to deliver SPSHK (Table 1). The study described herein focuses on the parent educators at the PAT affiliate sites and follows a prospective, longitudinal, cohort design. Parent educators will be invited to participate in provider-focused research during their onboarding (Figure 1). To be eligible, parent educators must be certified to deliver PAT, engaged in the Smart Parents Study, and have an active parent caseload. Parent educators who elect not to participate in provider research will still have the opportunity to be trained in SPSHK, invite parents to the Smart Parents Study, and deliver the SPSHK visit to families on their caseload.

**Table 1:** Provider training schedule.

Step	Year 1											
	Sept	Oct	Nov	Dec	Jan	Feb	Mar	Apr	May	Jun	Jul	Aug
Step 1				X	Y							
Step 2					X	Y						
Step 3						X	Y					
Step 4									X	Y		
Step 5											X	Y

X indicates the month in which parent educators at sites in the corresponding step will complete the T0 baseline assessment. Y indicates the month in which parent educators at sites in the corresponding step will complete SPSHK provider training and the T1 post-training assessment.

### Experimental procedures

Immediately following consent, parent educators who agree to participate will be asked to complete a one-time demographic questionnaire. In the 4 years following, participants will be asked to complete a 15-to-20-min online assessment at 6 timepoints: (T0) 1 month prior to being trained in the SPSHK curriculum; (T1) immediately post-training; and (T2-T4) annually thereafter for 3 years (Figure 1). All assessments will be administered in English via REDCap (Research Electronic Data Capture) (29). Participants will be emailed a unique link to each assessment at the appropriate time intervals, and follow-up emails reminding participants to complete the survey will be auto-sent every 3 days up to four times to promote retention. Participants will have a 6-week window to respond before being considered lost to follow-up for that timepoint. Those who miss the window for one assessment will still be invited to participate in the next. In conjunction with each of the annual assessments (T2-T4), participants will be invited to engage in 30-min audio-video recorded semi-structured interview via Zoom. These interviews will be offered in both English and Spanish, depending on the provider’s preference. Providers will receive an email 2 weeks prior to their respective T2, T3, and T4 timepoints to schedule the interview and will have an additional 4 weeks to complete the interview before they will be considered lost to follow-up for that timepoint. Parent educators will receive Amazon e-gift cards for their participation in the amount of $25 for T0 and T1 assessments, and $50 for each annual assessment and interview – totaling $200 for complete participation.

Organizational leadership at participating sites will be asked to complete a 15-min online assessment following their initial enrollment (Figure 1). These data will be used to contextualize providers’ responses within their work environments. Sites are compensated annually for their participation in the larger Smart Parents Study. Provider enrollment began in late October 2024. Study procedures and data collection are projected to continue through August of 2028.

### SPSHK training

All parent educators are onboarded to the Smart Parents Study shortly following site enrollment with a 30-min series of self-paced online video modules covering good clinical practice, CSA as a public health problem, and Smart Parents Study procedures. One month prior to their SPSHK training, parent educators will receive a unique link via email to complete an additional 1.5-h series of online video modules. This content will include an overview of the SPSHK curriculum, recordings of a mock SPSHK visit, and reminders about study procedures. The online modules will also include a live 2-h early interventionists orientation to child maltreatment workshop presented by study partners at the Hugh Lane Wellness Foundation.

Following these self-directed and online training efforts, members of the university-based research team will travel to each PAT site to lead a 4-h in-person SPSHK training. Parent educators will receive their copies of the Provider Guidebook (i.e., curriculum manual) and Parent Handbook. In alignment with social learning theory and best practices in provider training, the research team will explain how to navigate the provider guidebook, model delivery of the curriculum, and allow parent educators the time to practice delivering SPSHK in pairs. After completing all training elements, parent educators will record and submit a mock SPSHK visit conducted with a colleague, friend or family member (i.e., not a family on their caseload) to be reviewed by the university-based research team. The research team provides feedback on fidelity to the parent educator (e.g., effective and accurate presentation of material, constructive feedback to practice scenarios, inclusion of all curriculum components, time management) and once mastery is met, certification. Fidelity will continue to be monitored throughout the study with provider implementation forms and parent satisfaction surveys completed after every delivery of SPSHK. Sites will receive a one-time stipend of $1,000 to cover provider training time.

### Measures

#### Organizational

Sites engaged in the Smart Parents Study are similar to one another in that they are all affiliates in good standing with PAT National Center and deliver the PAT curriculum to families; however, sites may vary in size, case structure, leadership, climate, and funding. Measures assessing some of these site-level characteristics to be completed by the organizational leadership at each site will include the Implementation Leadership Scale (ILS) (30) and the Implementation Climate Scale (ICS) (31), both of which were validated with samples of US clinical mental health providers primarily specializing in family therapy and social work. The ILS is a 12-item assessment developed to evaluate organizational-level support of evidence-based practice. Items such as “I have removed obstacles to the implementation of evidence-based practice” and “I support employee efforts to use evidence-based practice” will be rated on a 5-point scale from (0) Not at all to (4) Very great extent. The ICS is an 18-item assessment designed to measure agency perceptions of new interventions. Items including “This team/agency selects staff open to new types of interventions” and “Clinicians who use evidence-based practices are held in high esteem in this team/agency” will be rated on the same scale as the ILS.

#### Provider

**Figure 2:**
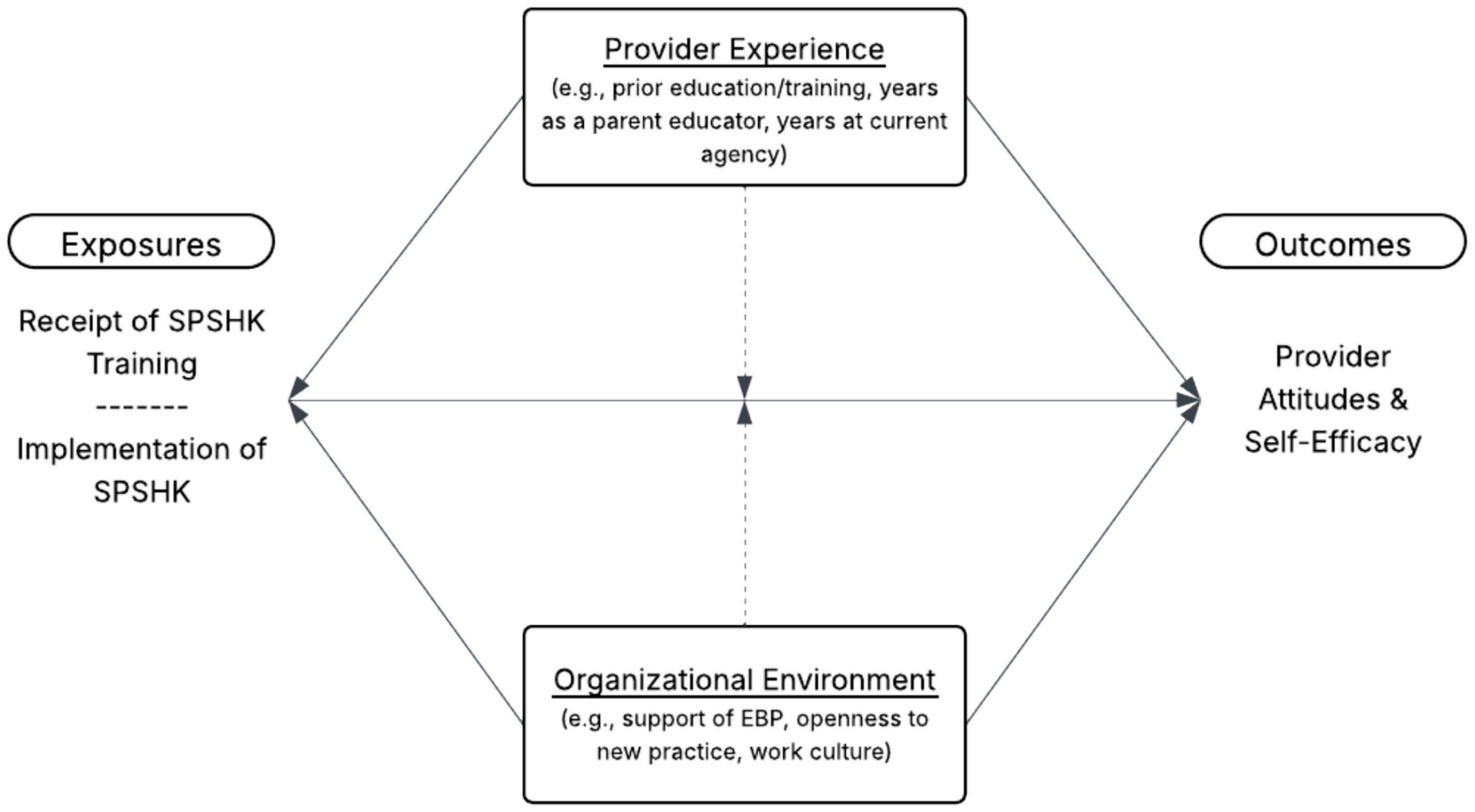
Theory of change.

Basic demographic information collected from parent educators includes age, race, ethnicity, education, religion, and years of experience both in the role of a parent educator and at their current site/agency. The pre-training assessment (T0) includes the Evidence Based Practice Attitudes Scale (EBPAS) (32) and a project measure specific to the implementation of SPSHK (Figure 2) (27). The 15-item EBPAS was designed to assess provider willingness to use and acceptability of evidence-based practice. Eight items ask participants to rate the extent to which they agree with statements such as “I am willing to try new types of therapy/interventions to help my clients” and “Research based treatments/interventions are not clinically useful” on a 5-point scale [(0) Not at all to (4) Very great extent]. Seven items focus on their likelihood of adopting an intervention that was new to them given a set of potential circumstances (e.g., “if it was required by your supervisor”; “if it was used by colleagues who were happy with it”) rated on the same 5-point scale. The EBPAS is only administered at T0.

The 11-item project measure was designed in a preliminary study with inspiration from Myers and colleagues and will be administered at all timepoints (i.e., T0-T4) (33). The purpose of this measure is to evaluate parent educators’ attitudes and self-efficacy pertaining to foundational concepts underlying the SPSHK curriculum (27). Due to small sample size in the study that this measure was originally developed for, it has not been validated. Pending sample size, we will explore a confirmatory factor analysis for latent constructs ‘attitudes’ and ‘self-efficacy.’ In the current study, ‘attitudes’ is operationally defined as the perceptions parent educators have toward the content and delivery modality of SPSHK (i.e., acceptability), both in general and pertaining to their own delivery of the curriculum. Items ask parent educators to respond to statements like “Discussing sex with parents or other adults goes against my personal beliefs” rated on a 5-point Likert scale from (1) Strongly disagree to (5) Strongly agree. ‘Self-efficacy’ is operationally defined in the current study as the extent to which a parent educator feels confident in their knowledge and skills to deliver SPSHK to families on their caseload. This concept is measured with items such as “I know how to respond if a parent asks me a question about their child’s sexual development” rated on the same 5-point scale. An additional 3 open text response items included with this measure at T0 and T1 timepoints ask participants to share thoughts, concerns, and what excites them about delivering the SPSHK visit.

At T1 (immediately following training), participants will complete a training satisfaction survey. This project-developed 25-item measure will be leveraged to gain insight on the ways in which current training practices can be better tailored to support future providers. Open text response items will ask participants to share their perceptions of SPSHK before and after training as well as their thoughts on training accessibility. Additional items (e.g., “I felt engaged while completing the online training modules;” “Talking through possible discussion points that may come up during a session with a parent was helpful”) will be rated on a 5-point Likert scale from (1) Strongly disagree to (5) Strongly agree. A final item will ask participants to rate how confident they feel that the training modules have prepared them to deliver SPSHK to families on their caseload on a 3-point Likert scale from (1) Not Confident to (3) Confident.

The semi-structured interview conducted alongside the project measure at T2-T4 will follow the RE-AIM framework (34, 35). Qualitative items will help the research team understand implementation outcomes, including the Reach, Effectiveness, Adoption, Implementation, and Maintenance of SPSHK as delivered by participating providers. Priority will be given to the latter three dimensions. Sample items include “In your opinion, how well do the parents who are participating in the Smart Parents Study reflect the parents you typically serve?” (Reach), “What changes, if any, have you observed in parents’ knowledge and behavior following the Smart Parents sessions?” (Effectiveness), “What factors influenced your decision to be a part of the Smart Parents Study?” (Adoption), “How closely did you follow the Smart Parents provider guidebook when delivering the sessions?” (Implementation), and “To what degree do you feel supported as a member of the Smart Parents Study team?” (Maintenance).

### Analytic plan

Given that this line of research is embedded within a larger trial (see Study Design), the sampling frame of providers was pre-determined by the number of parent participants needed to power the broader trial. Our primary modeling approach will use linear mixed-effects regression to fit the outcome ‘self-efficacy’ longitudinally over the five study time points (TP). We will include covariates to adjust for study wave (SW), and random intercepts at the provider- and site-level to account for repeat measurements and any potential clustering:


$$\begin{array}{c} \text{self efficacy}=\beta_{0}+\beta_{1}\times TP_{1}+\beta_{2}\times TP_{2}+\beta_{3}\times TP_{3}\\ +\beta_{4}\times TP_{4}+\sum_{j=1}^{5}\beta_{4+j}SW_{j}+\lambda_{i}+\kappa_{j}, \end{array}$$

where i indexes providers and j indexes sites; $\beta_{1}$ - $\beta_{4}$ represent the coefficients for the fixed effects for each time point; $\beta_{5}$ - $\beta_{9}$ , the fixed effects for study wave; and $\lambda_{i}$ and $\kappa_{j}$ represent the at the provider- and site-level random intercepts. We will report $\beta_{1}$ with 95% confidence intervals as our primary measure of the change in self-efficacy from baseline to TP_1_. The same modeling approach will be fit for the outcome ‘attitudes’.

To test for potential effect modification, we will add interaction terms with each moderator at each timepoint. Moderators will include study wave and provider and site characteristics as specified in Table 2. We anticipate minimal missing demographic information for parent educators. Our primary approach to address occasional skipped survey responses will be to use multiple imputation; as a sensitivity analysis, we will conduct best-case and worst-case single imputation (filling in all low scores and all high scores).

**Table 2:** Study outcomes.

Variables	Measure
Primary outcomes	
Self-efficacy	Project measure
Attitudes	Project measure
Moderators	
Study wave (i.e., step)	N/A
Demographics	Demographic survey
Experience	Demographic survey
Attitudes on EBP	EBPAS
Site-level characteristics	Organizational leadership survey (ILS; ICS)

We will track retention carefully and have implemented strategies to promote parent educator retention in both the provider-focused research as well as the broader Smart Parents Study. These strategies include an incremental incentive structure, regular implementation meetings and newsletters, and Smart Parents Study T-shirts, tote bags, and mugs at study milestones. We will explore the rate and pattern of attrition throughout the study, testing for any significant provider-level factors associated with loss to follow up. Our primary statistical method to address attrition if it arises will be to use the mixed-effects regression model above, which implicitly assumes missingness at random. As a sensitivity analysis, we will also fit a version of the model where timepoints with more than 30% attrition are dropped to understand whether results are robust to possible attrition bias.

Open-ended responses from the project measure will be coded in Excel leveraging a phenomenological approach. Qualitative data from the annual semi-structured interviews will be transcribed, deidentified, coded, and analyzed thematically in Dedoose by ≥2 members of the research team with 10% overlap and goal of 90% interrater reliability (36). The codebook will center study aims and be rooted deductively in alignment with the RE-AIM framework; however, we will also allow for inductive coding based on themes that may arise from participant responses (34, 35, 37). The principal investigator will code an additional 10% of interviews chosen at random evenly spread across participants in each study wave to promote reliability throughout.

#### Sample size justification

The target sample size of 100 parent educators was determined to address a different research aim. To achieve the sample size of parents needed to power the primary outcome for the Smart Parents Study, we anticipated that we would need to engage about 100 parent educators. With the target sample size for the current study, we will have 80% power to detect an effect size in the range *d* = 0.18–0.25 for the change in either self-efficacy or attitudes pre-post, assuming baseline self-efficacy and a test–retest correlation of 60–80% and a significance threshold of α=5% .

### Limitations

The methods described herein have several limitations worth noting due to the study’s nested structure. First, this study is largely dependent on the design and timeline of the parent-focused Smart Parents Study. For instance, delays in the Smart Parents Study (e.g., parent recruitment targets) may translate to delays in training and assessments for the provider study. Second, the sampling frame of providers is limited to those already engaged in the Smart Parents Study as a result of their PAT site’s voluntary participation, which introduces sampling bias. In addition, the number of PAT sites recruited to participate in the Smart Parents Study was determined based on what was needed to adequately power the parent-focused study, not the nested provider-focused study. Lastly, although providers are assured throughout their involvement that assessment responses are confidential, it should be noted that potential challenges related to providers’ discomfort or lack of familiarity with CSA-related content may have an impact on their initial and continued willingness to participate in the provider research component or the extent to which they share during assessments.

## Discussion

Rates of CSA have remained largely static in recent decades indicating a need for innovative approaches to prevention (38). The existing infrastructure of home visiting models fosters great promise for incorporation of CSA prevention content given the federal support, foundational subject-matter covered, and engagement of higher risk families (39). Further, parent educators are well-positioned to deliver this content to parents. Parent educators possess a strong foundation for best practices in parenting and child development, and are skilled in rapport building, which can create an opportunity for approaching and discussing sensitive topics with their clients (40). However, content related to CSA prevention has not been included in evidence-based home visiting program curricula and thus is novel to many home visitors. The Smart Parents Study provides the opportunity to learn not only from parents receiving SPSHK, but from the parent educators delivering it. The current study provides the opportunity for the home visiting field to understand provider acceptability and self-efficacy, as well as implementation barriers and facilitators for CSA prevention content delivery in existing home visiting programs. Translated into action, outcomes of the current study will inform best practices for supporting parent educators through training and implementation stages of SPSHK, bolstering future reach, fidelity, and effectiveness of implementation, and in turn, result in a public health impact on CSA. Additionally, learnings from this trial may apply to other home visiting enhancements such as modules designed to reduce secondhand smoke exposure (41) or healthy eating (42) among parents enrolled in home visiting programs, thereby maximizing public health impact.

## Ethics and dissemination

All study procedures have been approved by the New York University Institutional Review Board (FWA00006386) Institutional Review Board. Informed consent will be collected from all providers who wish to participate. Providers will be informed that their participation is completely voluntary and separate from their involvement in inviting parents and delivering SPSHK in the broader, Smart Parents Study. Providers are able to withdraw from their participation at any time. Any and all changes made to the current protocol will be submitted for ethical review prior to their integration.

Upon study completion, the research team anticipates disseminating findings through two channels, one being academic publication. An initial paper will seek to evaluate the experience of providers from pre-training to immediately post-training in the SPSHK curriculum, and throughout their delivery of SPSHK in the years following. Another paper will integrate effectiveness data from the Smart Parents Study (parent data) to look at between provider differences that may inform future training and supervisory efforts. In a second channel of dissemination, results and implications will be shared with the Home Visiting Applied Research Collaborative (HARC) so that other investigators working on sensitive topics with home visiting providers can learn about threats to implementation and methods to support its success.
